# Copy Number Analyses Identified a Novel Gene: *APOBEC3A* Related to Lipid Metabolism in the Pathogenesis of Preeclampsia

**DOI:** 10.3389/fcvm.2022.841249

**Published:** 2022-05-16

**Authors:** Nan Liu, Yu-Na Guo, Xiao-Jin Wang, Jue Ma, Yun-Ting He, Fang Zhang, Hao He, Jin-Liang Xie, Xu Zhuang, Meng Liu, Jian-Hua Sun, Yan Chen, Jian-Hua Lin, Li-Kun Gong, Bing-Shun Wang

**Affiliations:** ^1^School of Pharmacy, University of Chinese Academy of Sciences, Beijing, China; ^2^State Key Laboratory of Drug Research, Center for Drug Safety Evaluation and Research, Shanghai Institute of Materia Medica, Chinese Academy of Sciences, Shanghai, China; ^3^Department of Obstetrics, International Peace Maternity and Child Health Hospital, Shanghai Jiao Tong University School of Medicine, Shanghai, China; ^4^Department of Biostatistics, Clinical Research Institute, Shanghai Jiao Tong University School of Medicine, Shanghai, China; ^5^School of Renji Clinical Medicine, Renji Hospital, Shanghai Jiao Tong University School of Medicine, Shanghai, China; ^6^Department of Obstetrics and Gynecology, Renji Hospital, Shanghai Jiao Tong University School of Medicine, Shanghai, China

**Keywords:** preeclampsia, *APOBEC3A*, lipid metabolism, copy number variation, genetics

## Abstract

**Background:**

Preeclampsia is a heterogeneous and complex disease with its pathogenesis mechanism not fully elucidated. A certain subset of patients with preeclampsia exhibit disturbances in lipid metabolism before clinical symptoms. Moreover, there is a tendency for preeclampsia to run in families. Whether genetic factors play a role in abnormal lipid metabolism during the incidence of preeclampsia has not been well investigated.

**Methods:**

Preeclampsia patients (*n* = 110) and healthy age- and gravidity-matched pregnant women (*n* = 110) were enrolled in this study. Peripheral blood specimens were used for genomic analysis (*n* = 10/group) or laboratory validation (*n* = 100/group). We retrospectively obtained the baseline clinical characteristics of 68 preeclampsia patients and 107 controls in early pregnancy (12–14 gestational weeks). Correlation analyses between differential genes and baseline lipid profiles were performed to identify candidate genes. *In vitro* and *in vivo* gain-of-function models were constructed with lentivirus and adeno-associated virus systems, respectively, to investigate the role of candidate genes in regulating lipid metabolism and the development of preeclampsia.

**Results:**

We observed that preeclampsia patients exhibited significantly elevated plasma TC (*P* = 0.037) and TG (*P* < 0.001) levels and increased body mass index (*P* = 0.006) before the disease onset. Within the region of 27 differential copy number variations, six genes potentially connected with lipid metabolism were identified. The aberrant copies of *APOBEC3A*, *APOBEC3A_B*, *BTNL3*, and *LMF1* between preeclampsia patients and controls were verified by quantitative polymerase chain reaction. Especially, *APOBEC3A* showed a significant positive correlation with TC (*P* < 0.001) and LDL (*P* = 0.048) in early pregnancy. Then, our *in vitro* data revealed that overexpression of *APOBEC3A* disrupted lipid metabolism in HepG2 cells and affected both cholesterol and fatty acid metabolisms. Finally, *in vivo* study in a hepatic-specific overexpressed *APOBEC3A* mouse model revealed abnormal parameters related to lipid metabolism. Pregnant mice of the same model at the end of pregnancy showed changes related to preeclampsia-like symptoms, such as increases in sFlt-1 levels and sFlt-1/PLGF ratios in the placenta and decreases in fetal weight.

**Conclusion:**

Our findings established a new link between genetics and lipid metabolism in the pathogenesis of preeclampsia and could contribute to a better understanding of the molecular mechanisms of preeclampsia.

## Graphical Abstract

Identification and validation of APOBEC3A’srole in regulating lipid metabolism in PE. 
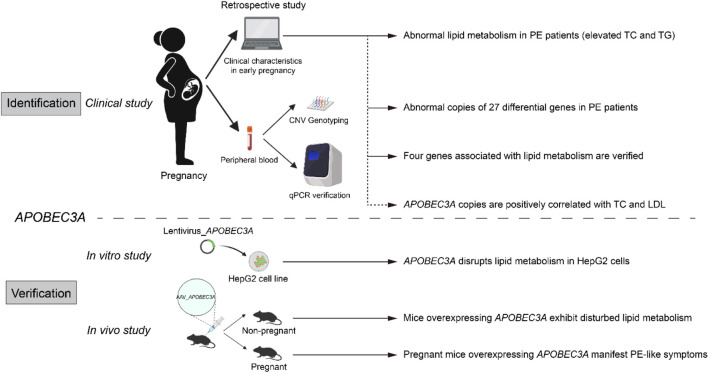

## Introduction

Preeclampsia (PE) is a pregnancy-specific complication mainly characterized by hypertension and proteinuria and remains one of the leading causes of morbidity and mortality in pregnant women and fetuses (1). Currently, the incidence of PE in developed countries is about 1.3–6%, which is even higher in developing countries (2). PE is a heterogeneous and complex disease caused by multiple factors, including heredity and environment (3). However, the underlying mechanism remains elusive due to diverse races, geography, and other factors (4).

An emerging body of research has demonstrated that abnormalities in lipid metabolism may be connected with the etiology of PE, with certain patients manifesting lipid metabolism disturbances before the onset of clinical symptoms (5–7). The lipid profiles are clinically accessible parameters. We have previously reported that triglycerides (TG) and low-density lipoprotein cholesterol (LDL) are independent risk factors for PE, while high-density lipoprotein cholesterol (HDL) is an independent protective factor (8). Therefore, elucidating the relationship between abnormal lipid metabolism and the pathogenesis of PE can guide the early identification of high-risk individuals for PE.

Previous findings have revealed that PE is relatively common among daughters and sisters of women with PE (9, 10), indicating genetic factors play an essential role in the development of PE. Most studies on genetic factors in PE are based on expression profiles, including mRNA and protein levels, making it challenging to clarify the causal relationship between these factors and the onset of PE.

To elucidate the mechanistic link between abnormal lipid metabolism and genetics in the pathogenesis of PE, we analyzed the genomes of clinical specimens with DNA microarrays and performed laboratory validation. Additionally, we retrospectively collected clinical characteristics of pregnant women in early pregnancy (12–14 gestational weeks) and performed the correlation analysis of lipid profiles at baseline with the candidate genes. Furthermore, we constructed *in vitro* and *in vivo* gain-of-function models to explore the role of candidate genes in regulating lipid metabolism and participating in the development of PE.

## Materials and Methods

### Clinical Study Design and Oversight

**FIGURE 1:**
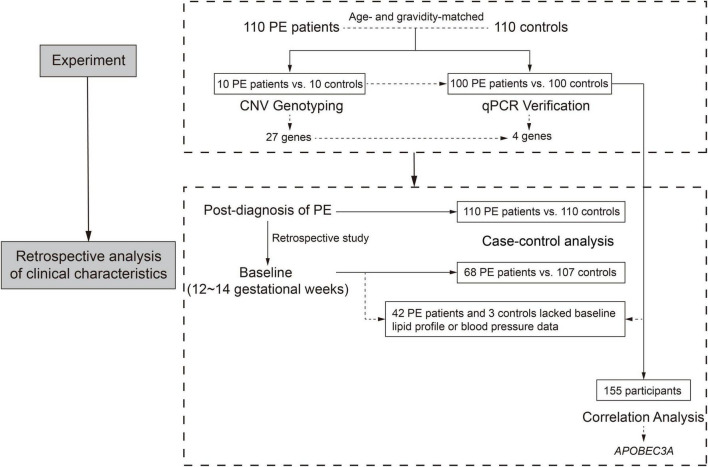
Study flow chart.

From October 2013 to December 2017, we recruited 220 participants, including 110 PE patients and 110 controls, from two institutes in Shanghai, China (Department of Obstetrics, International Peace Maternity and Child Health Hospital, and Department of Obstetrics, Renji Hospital, Shanghai Jiao Tong University School of Medicine). The case-control study flow chart is displayed in Figure 1. Fasting peripheral blood specimens were collected before delivery. The inclusion criteria for the PE group were pregnant women with hypertension (≥ 140 mmHg systolic and ≥ 90 mmHg diastolic blood pressure), proteinuria higher than 0.3 g/d or qualitatively + above. In contrast, healthy age- and gravidity-matched pregnant women with normotensive pre-pregnancy and pregnancy and without obstetric complications as the control group.

Table 1 lists clinical features at the patients’ initial diagnosis of PE. We retrospectively collected clinical characteristics of subjects at their initial routine prenatal examination in early pregnancy (12–14 gestational weeks), which is listed in Table 2. Since the Department of Obstetrics at Renji Hospital is a consultation and resuscitation center for maternal near-miss in Shanghai, some PE patients in this hospital were referred from other institutions, so the baseline data of some subjects in early pregnancy (12–14 weeks of gestation) are missing. The study was conducted in accordance with the Declaration of Helsinki and was approved by the Medical Research Ethics Committee of International Peace Maternity and Child Health Hospital (the Ethics Committee Approval Protocol No. 5 of 28 February 2014). All subjects provided broad informed consent for the research use of their biological samples.

**TABLE 1:** Clinical characteristics of participants at diagnosis.

Variables	PE patients (*n* = 110)	Controls (*n* = 110)	*P*-value
Age, years	31.00 (8.00)	31.00 (5.25)	0.205
SBP, mmHg	147.00 (16.00)	119.00 (11.00)	< 0.001^a^
DBP, mmHg	94.00 (12.00)	75.00 (9.00)	< 0.001^a^
Gestational age at delivery, week	37.00(3.20)^b^	38.80 (1.30)	< 0.001^a^
Proteinuria, g/24 h	0.42 (0.95)	0.06 (0.05)	< 0.001^a^
Fetal weight, g	2830.00(1170.00)^b^	3307.50 (428.75)	< 0.001^a^

*PE, preeclampsia; SBP, systolic blood pressure; DBP, diastolic blood pressure. Variables were expressed as median (IQR). The p-values were calculated by the unpaired two-tailed Mann-Whitney U-test.*

*^a^P ≤ 0.05; ^b^Excluding 7 stillbirths due to early termination of pregnancy in patients with severe PE.*

**TABLE 2:** Clinical characteristics of participants in early pregnancy (12–14 gestational weeks).

Variables	PE patients (n = 68)	Controls (*n* = 107)	*P*-value
Body mass index, kg/m^2^	23.00 (4.00)	21.40 (3.10)	0.006^a^
SBP, mmHg	110.00 (14.00)	110.00 (13.00)	0.102
DBP, mmHg	70.00 (11.00)	70.00 (4.50)	0.240
TC, mmol/L	4.62 (0.93)	4.46 (0.70)	0.037^a^
TG, mmol/L	1.61 (1.13)	1.27 (0.64)	< 0.001^a^
HDL, mmol/L	1.82 (0.69)	1.81 (0.38)	0.839
LDL, mmol/L	2.71 (0.85)	2.48 (0.64)	0.056

*PE, preeclampsia; SBP, systolic blood pressure; DBP, diastolic blood pressure; TC, total cholesterol; TG, triglyceride; HDL, high-density lipoprotein; LDL, low-density lipoprotein.*

*Variables were expressed as median (IQR). The p-values were calculated by the unpaired two-tailed Mann-Whitney U-test. ^a^P ≤ 0.05.*

### Copy Number Variation Genotyping and Data Analysis

Genomic DNA was extracted from the peripheral blood (*n* = 10/group) of pregnant women and detected using Affymetrix CytoScan HD Cytogenetics Chips according to the manufacturer’s recommended protocol. Quality control analysis indicated that all microarrays were qualified, except one was judged as male by the Chromosome Analysis Suite software for unclear reasons (Supplementary Table 1). Therefore, we excluded this sample from the analysis of the microarray data.

The raw data was processed by Chromosome Analysis Suite analysis software from Affymetrix to obtain all the CNVs, including duplications and deletions. Given the small sample size for microarray analysis, we opted for CNVs that occurred with a frequency difference of more than 30% between the two groups. These CNVs did not overlap with the high-frequency CNV regions of ordinary individuals in the Database of Genomic Variants^1^ as differential CNVs. The differential genes within the region of differential CNVs were given gene annotation by the Gene Ontology (GO) database. The GO function enrichment analysis was employed to obtain the special functions for these genes using Fisher’s exact test and χ2 test with the threshold of *P* < 0.05 and FDR < 0.25.

### Verification of Gene Copy Numbers and Clinical Correlation Analysis

Total genomic DNA was extracted from peripheral blood specimens (*n* = 100/group) of pregnant women using the AxyPrep Multisource Genomic DNA Miniprep Kit (Axygen Scientific) following the manufacturer’s instructions. The genes linked to lipid metabolism were verified by quantitative polymerase chain reaction (qPCR), and *GAPDH* was used as an endogenous control at the DNA level. Details of the primer sequences are shown in Supplementary Table 2. In this study, correlations between candidate genes and clinical lipid profiles were analyzed by SPSS Statistics 25, using Pearson’s correlation test.

### Establishment of Lipid Accumulation Model *in vitro*

HepG2 (ATCC) cell line was cultured in DMEM medium (Gibco) supplemented with 10% fetal bovine serum (Gibco), 1% Penicillin-Streptomycin (Thermo Fisher Scientific) at 37^°^C in a 5% CO2 atmosphere. We infected cells with lentivirus carrying *APOBEC3A* _Flag or negative control and screened the cells using puromycin (Thermo Fisher Scientific) to obtain a pool of cells overexpressing the *APOBEC3A* gene (or negative control). DMEM medium with 1 mM oleic acid (Sigma) served as the fat accumulation inducer for the cells. Cell cultures were collected after being starved overnight and treated with the fat accumulation inducer for 24 h. The intracellular lipid content was determined by a biochemical analyzer (Roche). The results were normalized to the total protein concentration.

### Adeno-Associated Virus-Mediated Overexpression of *APOBEC3A* in Mice

The coding sequence of human *APOBEC3A*, the 3 × flag tag, and a green fluorescent protein were simultaneously subcloned into the AAV2/8 plasmid (AAV_*APOBEC3A*) under the control of the thyroxine-binding globulin promoter, a hepatic-specific promoter. This serotype is known to have a stable expression in the liver after injection (11). A virus containing green fluorescent protein alone under control of the thyroxine-binding globulin promoter was used as a control virus (AAV_Mock).

Separate groups of 6- to 8-week-old wild-type, C57BL/6J female mice (*n* = 14/group) were injected intraperitoneally with 1.5 × 10^10^ vector genomes/mouse of the relevant vectors. Mice were fed conventional chow throughout the study. They were sacrificed to investigate the impact of *APOBEC3A* on lipid metabolism (*n* = 6/group) or mated to explore the effect of the *APOBEC3A* gene on pregnant mice (*n* = 8/group, a positive vaginal plug was marked as gestation day 0, pregnant mice were sacrificed on gestation day 18) at 5 weeks (35 days) after AAV administration. Mice were fasted for 4 h, anesthetized with isoflurane, and whole blood was collected by cardiac puncture into BD Microtainer EDTA collection tubes. Plasma samples were used for lipid analysis and assayed using a biochemical analyzer (Roche). Liver and placenta specimens were obtained and immediately fast-frozen in nitrogen and stored at −80^°^C for subsequent testing. Fetuses were delivered by cesarean section, and their weights were measured. We performed all animal procedures according to protocols approved by the Institutional Animal Care and Use Committee of Shanghai Institute of Materia Medica, Chinese Academy of Sciences.

### RNA-Seq and Cluster Analysis

Total RNA was extracted from HepG2 cells (*n* = 3/group) and used for RNA-seq analysis. cDNA library construction and sequencing were performed by the Shanghai Majorbio Bio-Pharm Technology Co., Ltd. using the majorbio cloud platform. High-quality reads were aligned to the human reference genome (GRCh38) using Bowtie2. We normalized the expression level of each gene to the fragment of the exon model per million mapped reads (FPKM) based on the expectation-maximization method. NOISeq method was used to screen out differentially expressed genes between the two groups with a fold change ≥ of 1.5. Function and pathway annotation and enrichment analysis were based on the GO database and the Kyoto Encyclopedia of Genes and Genomes database.

We obtained the list of genes connected with cholesterol homeostasis and fatty acid metabolism from the Gene Set Enrichment Analysis website. The FPKM of these genes were logarithmically (fold-change) converted, and we visualized the differences between the two groups through heatmap, which was performed by GraphPad Prism V.8 software.

### Reverse Transcription-Polymerase Chain Reaction and Immunoblot Analysis

Cell cultures (*n* = 3/group) or liver tissues (*n* = 6/group) were lysed in TRIzol (Invitrogen), and total RNA was extracted according to the manufacturer’s instructions. cDNA was synthesized using PrimeScript™ RT Master Mix (Takara). A reverse transcription-polymerase chain reaction was performed using PowerUp™ SYBR™ Green Master Mix (Thermo Fisher Scientific) on an Applied Biosystems 7500 Fast Real-Time PCR System (Thermo Fisher Scientific). Primer sequences for human cells are listed in Supplementary Table 3 (Supplementary Table 4 is for mouse tissues). All results were normalized to *GAPDH* expression and calculated using the comparative CT (ΔΔCT) method.

Cell cultures (*n* = 3/group) were lysed with RIPA lysis buffer (Thermo Fisher Scientific). Protein lysates were separated on 10% SDS-PAGE gels and electrophoretically transferred to polyvinylidene fluoride membranes (Millipore). The membranes were blocked with 5% skim milk and incubated overnight at 4^°^C with mouse monoclonal anti-FLAG^®^ M2 (Sigma) as primary antibodies. Then, membranes were incubated with horseradish peroxidase-conjugated secondary antibody (Goat anti-rabbit, or -mouse IgG, Jackson ImmunoResearch). Protein band chemiluminescence was detected using an ECL Plus immunoblot detection system (Millipore). We performed densitometric analysis on low-exposure images with ImageJ (National Institutes of Health). Rabbit polyclonal anti-β-Tubulin antibody (Proteintech) was used as a loading control to confirm equivalent protein loading on the same membrane.

### Immunohistochemistry Analysis

Fresh liver tissue samples were fixed in 4% paraformaldehyde and embedded in paraffin. We then sectioned them into 4-μm-thick sections for immunohistochemical staining. The primary antibodies used in this assay were rabbit monoclonal IgG [EPR25A]-isotype control (Abcam) and rabbit polyclonal to APOBEC3A (Abcam). Immunostaining was performed according to the standard protocol. Then, labeling was visualized using the diaminobenzidine method. Counterstaining was performed using Mayer’s hematoxylin.

### Enzyme-Linked Immunosorbent Assay

Approximately 25 mg of the liver (*n* = 6/group) or placental tissue (*n* = 8/group) was weighed and made into a 10% saline tissue homogenate, centrifuged at 4^°^C for 10 min at 3,000 g to obtain the supernatant for subsequent assays. TG or total cholesterol (TC) levels in the liver were measured by a biochemical analyzer (Roche). Placental growth factor (PLGF) or soluble fms-like tyrosine kinase-1 (sFlt-1) concentrations in plasma or placenta were measured by mouse PLGF or sFlt-1 Enzyme-linked immunosorbent assay kits (R&D Systems). The results were normalized by tissue mass.

### Untargeted Relative Quantitative Lipidomics

Lipids from liver specimens (*n* = 6/group) were extracted according to the methyl tert-butyl ether method (12). Samples were separated and analyzed using an ultra-performance liquid chromatography system (Waters) equipped with a Q-Exactive hybrid quadrupole-Orbitrap mass spectrometer (Thermo Fisher Scientific). Lipid lists were obtained by annotation using Lipid Search software, and differential lipids were identified by *t*-test and orthogonal partial least squares discrimination analysis test. The Beijing Genomics Institute performed this assay.

### Statistical Analysis

Data were expressed as mean (*SD*) or median (IQR) when appropriate. Comparisons of continuous variables between two groups were made by unpaired two-tailed Student’s *t*-test or Mann-Whitney *U*-test when variables were normally or non-normally distributed, respectively. Statistical tests were performed by GraphPad Prism V.8. Correlation analysis was conducted by SPSS Statistics 25. *P* ≤ 0.05 (*) was considered statistically significant. Other statistical analysis methods used have been described separately in the respective methods section.

Graphical abstract was created using graphics from www.Biorender.com.

## Results

### Preeclampsia Patients Showed a Trend of Abnormal Lipid Metabolism Before the Onset of the Disease

Compared with the control group, patients with PE presented with elevated blood pressure, increased proteinuria, decreased gestational age at delivery, and fetal growth restriction (Table 1), consistent with previous reports (13). The clinical characteristics of 68 PE patients and 107 controls in early pregnancy (12–14 gestational weeks) were retrospectively obtained, as shown in Figure 1 and Table 2. The findings showed that those pregnant women who subsequently developed PE exhibited distinctly elevated plasma TC (*P* = 0.037) and TG (*P* < 0.001) levels before the disease onset and had significantly increased body mass index (*P* = 0.006). However, their blood pressure (systolic blood pressure, *P* = 0.102; diastolic blood pressure, *P* = 0.240) did not differ substantially in early pregnancy compared to controls. These findings indicate that PE patients tend to have abnormal lipid metabolism before the onset of the disease, but the causative mechanisms have not been elucidated.

### Differential Genes Between Two Groups Were Enriched in Functions Related to Metabolic Processes

**FIGURE 2:**
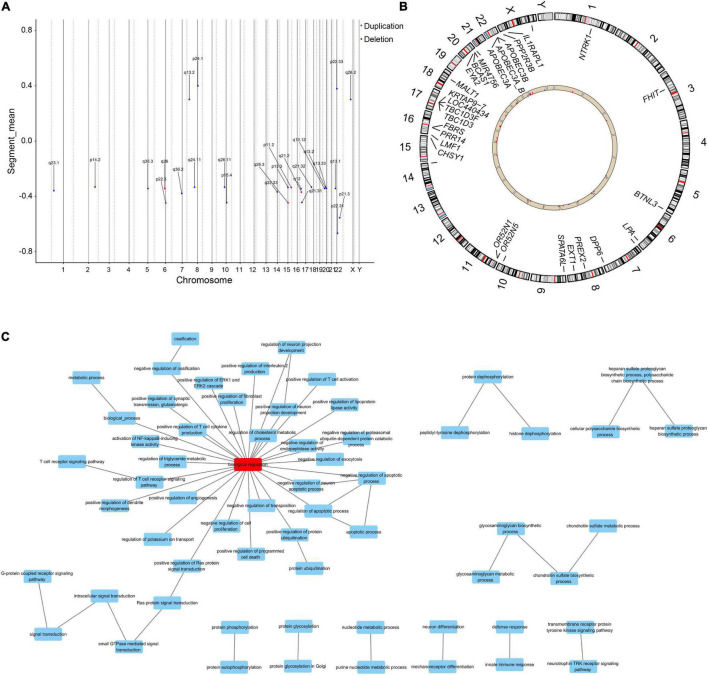
The differential genes linked to aberrant CNVs are enriched in metabolism-related biological functions. **(A)** Distributions of aberrant CNVs on each chromosome. Red and blue dots indicate whether the type of the CNV is duplication or deletion, respectively. **(B)** Distribution of differential genes within the region of differential CNVs in the genome. Red columns indicate that the copy number of this gene is increased in PE patients compared to controls, and blue columns indicate a decrease. **(C)** Differential genes were enriched in functions associated with lipid and cholesterol metabolism, immunity, and development.

From the genomic analysis of 10 PE patients and 10 controls (clinical characteristics are shown in Supplementary Table 5), we obtained 27 differential CNVs, including 22 deletions and 5 duplications (Figure 2A). The identified variants ranged in size from 1,409 base pairs to nearly 151.7 kb (median = 10.8 kb), and these CNV regions contained 27 differential genes (Figure 2B and Supplementary Table 6). GO functional enrichment analysis revealed that the biological functions of these differential genes were enriched in response to nutrient levels, protein hydrolysis, developmentally programmed cell death, and regulation of triglyceride/cholesterol/lipoprotein metabolism (Figure 2C).

### Aberrant Copies of Genes Linked to Lipid Metabolism Were Verified in 100 Preeclampsia Patients and 100 Controls

**FIGURE 3:**
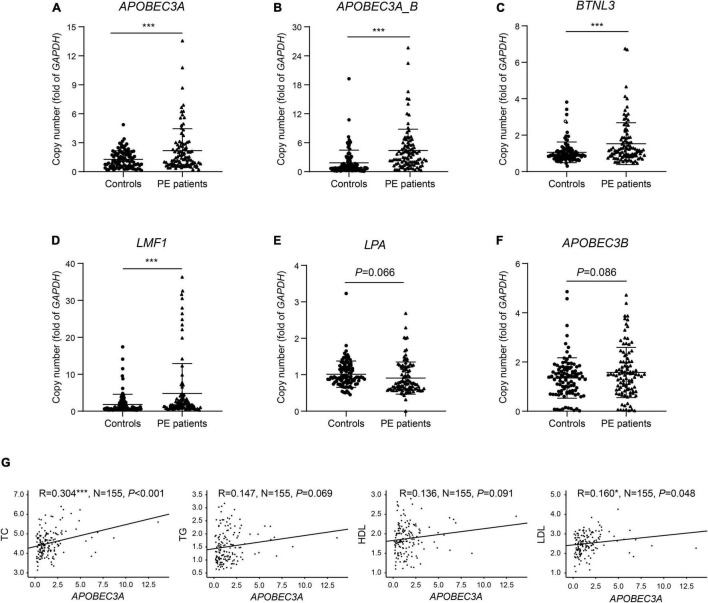
Verification of copies for lipid metabolism-related genes and correlation of copies of *APOBEC3A* with lipid profiles. The copy numbers of **(A)** *APOBEC3A*, **(B)** *APOBEC3A_B*, **(C)** *BTNL3*, **(D)** *LMF1*, **(E)** *LPA*, **(F)** *APOBEC3B* in PE patients and controls (*n* = 100/group). **(G)** Correlation of plasma lipid profiles with *APOBEC3A* copies (*n* = 155). The unpaired two-tailed Student’s *t*-test determined statistical significances between two groups. The Pearson correlation coefficient analyzed the correlations. Data were represented as the mean ± SEM. ***P < 0.001, *P < 0.05. PE, preeclampsia.

We identified six genes in the differential CNV regions that may be connected with lipid metabolism. The *LPA* gene located in the 6q26 region from 161.032 to 161.047 Mb was reported in previous research on PE (14–17), and we detected this duplication in one case (10%) and four controls (44.4%). *APOBEC3A*, *APOBEC3A_B*, and *APOBEC3B* in 22q13.1 from 39.350 to 39.390 Mb were detected in one case (10%) and four controls (44.4%). The *BTNL3* gene in the 5q35.3 region from 180.379 to 180.431 Mb, with this deletion detected in one case (10%) and four controls (44. 4%). The *LMF1* gene in the 16p13.3 region from 1.002 to 1.003 Mb, with this duplication detected in no one case and three controls (33.3%). These four regions of differential CNVs were confirmed by qPCR with significantly increased copy numbers of *APOBEC3A*, *APOBEC3A_B*, *BTNL3*, and *LMF1* in PE patients compared with controls (Figures 3A–D). In contrast, the decrease in copies of *LPA* and the increase in copies of *APOBEC3B* were not statistically significant (Figures 3E,F).

### *APOBEC3A* Showed a Significant Positive Correlation With Total Cholesterol and Low-Density Lipoprotein Cholesterol in Human Subjects

To identify the predisposing genes causing lipid metabolism disturbance in PE patients, we collected clinical lipid profiles from these participants’ initial prenatal examination (12–14 gestational weeks) and analyzed the correlation between lipid profiles and the copy number of these candidate genes. The results indicated significant positive correlations between the copy number of *APOBEC3A* and both TC (*P* < 0.001) and LDL (*P* = 0.048) levels in serum (Figure 3G). In contrast, we did not find significant correlations between other candidate genes and clinical lipid profiles here, except for copies of both *BTNL3* (*P* = 0.012) and *LPA* (*P* = 0.006), which were significantly positively correlated with HDL levels (Supplementary Figures 1A–E). For the first time, we report an interesting finding that the *APOBEC3A* gene may play a role in regulating lipid metabolism and be related to the pathogenesis of PE. Subsequently, we investigated how the novel gene *APOBEC3A* participates in regulating lipid metabolism and the pathogenesis of PE.

### *APOBEC3A* Disrupted Lipid Metabolism in HepG2 Cells *in vitro*

**FIGURE 4:**
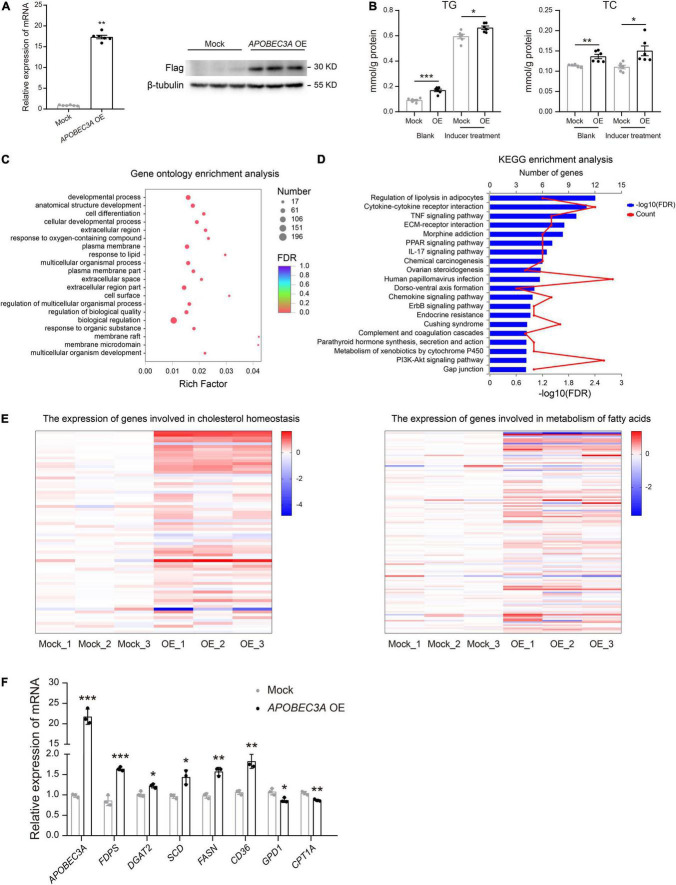
HepG2^**APOBEC*3A*^^OE^ exhibited perturbed cholesterol and fatty acid metabolism. **(A)** HepG2 cell lines over-expressing *APOBEC3A* were constructed. **(B)** TG and TC levels in the lysates of HepG2^**APOBEC*3A*^^OE^ and mock cells (*n* = 6/group). **(C)** Differential genes between HepG2^**APOBEC*3A*^^OE^ and mock cells were enriched in functions of respond to lipid. **(D)** Differential genes between HepG2^**APOBEC*3A*^^OE^ and mock cells were enriched in pathways of the regulation of lipolysis in adipocytes. **(E)** The expression of genes connected with cholesterol homeostasis and metabolism of fatty acids in HepG2^**APOBEC*3A*^^OE^ and mock cells (*n* = 3/group). **(F)** The mRNA expression of multiple genes related to cholesterol and fatty acid metabolism in HepG2^**APOBEC*3A*^^OE^ and mock cells (*n* = 3/group). The unpaired two-tailed Student’s *t*-test determined statistical significances between two groups. Data were represented as the mean ± SEM. ***P < 0.001, **P < 0.01, *P < 0.05. OE, HepG2^**APOBEC*3A*^^OE^ cells; Mock, HepG2 mock cells.

The liver is recognized as an essential metabolic organ in humans (18). To explore the underlying mechanism of *APOBEC3A* in lipid metabolism regulation, we constructed an *in vitro* model of HepG2 (HepG2^**APOBEC*3A*^^OE^), a human hepatocellular carcinoma cell line overexpressing *APOBEC3A* (Figure 4A). Both TC and TG levels were distinctly upregulated in HepG2^**APOBEC*3A*^^OE^ cells compared to mock cells treated with or without fat accumulation inducer (Figure 4B). RNA-seq analysis revealed that the differentially expressed genes in HepG2^**APOBEC*3A*^^OE^ were enriched in the biological function of response to lipid (Figure 4C) and the pathway of regulation of lipolysis in the adipocytes (Figure 4D). Furthermore, hallmark gene sets related to cholesterol homeostasis and metabolism of fatty acids in HepG2^**APOBEC*3A*^^OE^ (Figure 4E) showed distinct differences compared to the mock cells. The genes linked to cholesterol biosynthesis (*FDPS*, *DGAT2*) and fatty acid biosynthesis (*SCD*, *FASN*), as well as fatty acid absorption (*CD36*), were upregulated in HepG2^**APOBEC*3A*^^OE^. On the other hand, the genes associated with cholesterol catabolism (*GPD1*) and fatty acid β-oxidation (*CPT1A*) were downregulated (Figure 4F). These findings indicate that *APOBEC3A* may be involved in both cholesterol and fatty acid metabolic processes.

### Mice With Hepatic-Specific Overexpression of *APOBEC3A* Exhibit Disturbed Lipid Metabolism

**FIGURE 5:**
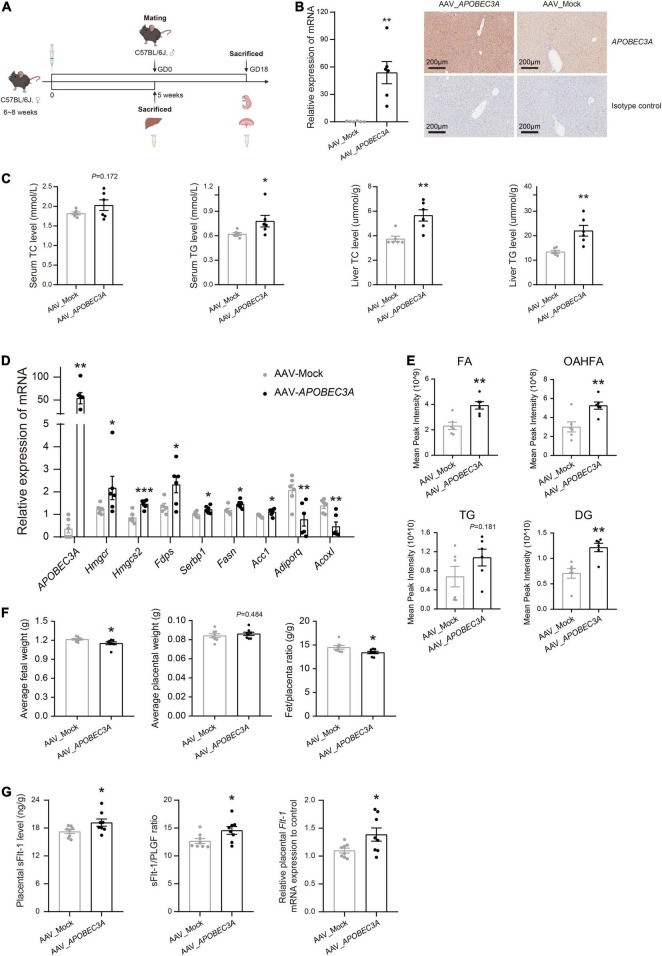
Hepatic-specific overexpression of *APOBEC3A* in mice manifested abnormal lipid metabolism and PE-like symptoms. **(A)** Experimental design and timeline for the *in vivo* experiment in mice. **(B)** The mRNA and protein expression of *APOBEC3A* gene in the liver of non-pregnant mice injected with AAV_*APOBEC3A* or AAV_Mock (*n* = 6/group). **(C)** TC and TG levels in plasma and liver of mice injected with AAV_*APOBEC3A* or AAV_Mock at 5 weeks after AAV administration (*n* = 6/group). **(D)** The mRNA expression of multiple genes related to cholesterol and fatty acid metabolism in mice injected with AAV_*APOBEC3A* or AAV_Mock (*n* = 3–6/group). **(E)** Levels of FA, OAHFA, TG, and DG metabolites in mice injected with AAV_*APOBEC3A* or AAV_Mock (*n* = 6/group). **(F)** The average weight of fetuses and placentas in pregnant mice injected with AAV_*APOBEC3A* or AAV_Mock (*n* = 8/group). **(G)** The protein and mRNA levels of sFlt-1 in the placenta of pregnant mice injected with AAV_*APOBEC3A* or AAV_Mock (*n* = 8/group). The unpaired two-tailed Student’s *t*-test determined statistical significances between two groups. Data were represented as the mean ± SEM. ***P < 0.001, **P < 0.01, *P < 0.05. FA, fatty acids; OAHFA, (O-acyl)-1-hydroxy fatty acids; TG, triglycerides; DG, diacylglycerol. GD, day of gestation.

To explore the potential roles of the *APOBEC3A* gene in regulating lipid metabolisms and participation in the pathogenesis of PE *in vivo*, we injected AAV_*APOBEC3A* and AAV_Mock intraperitoneally in adult C57BL/6J female mice (Figures 5A,B and Supplementary Figure 2). The first important finding was that TC and TG levels were elevated in both plasma and liver of *APOBEC3A* over-expressing mice (Figure 5C). Moreover, the expression of genes related to cholesterol biosynthesis (*Hmgcr*, *Hmgcs2*, *Fdps*) and fatty acid biosynthesis (*Srebp1*, *Fasn*, *Acc1*) were increased. In contrast, the expression of genes linked to fatty acid β-oxidation (*Adiporq*, *Acoxl*) was decreased in the liver of *APOBEC3A* over-expressing mice (Figure 5D). Lipidomics indicated that fatty acids, (O-acyl)-1-hydroxy fatty acids, triglycerides, and diacylglycerol were elevated in mice over-expressing *APOBEC3A* (Figure 5E and Supplementary Figure 3A). Moreover, pathway enrichment analysis indicated that differential lipids were enriched in glycerophospholipid metabolism, adipocytokine signaling pathway, fat digestion and absorption, regulation of lipolysis in adipocytes, and cholesterol metabolism (Supplementary Figure 3B). These results imply that *APOBEC3A* may play a significant role in the biosynthesis of fatty acids and triglycerides.

### Pregnant Mice With Hepatic-Specific Overexpression of *APOBEC3A* Exhibit Preeclampsia-Like Symptoms

Fetuses in the AAV_*APOBEC3A* group had decreased fetuses/placenta ratios, which appeared to be driven by a significant reduction in fetal weight (Figure 5F), showing intrauterine growth restriction, a common clinical manifestation of PE. The sFlt-1/PLGF ratio is a well-known marker for the short-term prediction of PE (19, 20). We measured the levels of PLGF and sFlt-1 in mouse placenta homogenates and serum by ELISA. The protein and mRNA levels of sFlt-1 and the sFlt-1/PLGF ratio were significantly increased in the placenta of the AAV_*APOBEC3A* group compared with the AAV_Mock group (Figure 5G). In contrast, there was no substantial change in both protein and mRNA levels of PLGF in placental tissues (Supplementary Figure 4A). Besides, these changes mentioned above were not observed in serum samples (Supplementary Figure 4B). These findings indicate that *APOBEC3A* may be involved in the pathogenesis of PE.

## Discussion

Overall, our study identified 27 aberrant CNVs related to PE, including 22 deletions and 5 duplications. The differential genes in these CNV regions were enriched mainly in metabolism-related biological functions. Moreover, aberrant copies of *APOBEC3A*, *APOBEC3A_B*, *BTNL3*, and *LMF1* were validated in our cohort, especially the copy number of *APOBEC3A* was markedly correlated with clinical lipid parameters of human subjects in early pregnancy. To our best knowledge, we are the first to report that *APOBEC3A* plays a role in regulating lipid metabolism and participating in the pathogenesis of PE.

Despite the growing attention to the pathogenesis of PE, there are still few reports investigating its pathogenesis related to CNV, i.e., duplications or deletions of genomic segments greater than 1 kb in length (21). Although most genome studies have focused on single nucleotide variants (SNVs), CNVs cover more bases than SNVs and may have more potent effects on gene expression and structure (22, 23). Zhao et al. (24) analyzed CNVs from a US cohort and identified two relevant genes, *PDXDC1* and *PSG11*. However, the hypothesis of them being involved in the pathogenesis of PE was not fully supported by further laboratory studies. The above two genes have no overlap with our findings, which may be interpreted as a difference in the genetic background of the two study cohorts.

Abnormal lipid metabolism in pregnant women has been reported to be possibly connected with the pathogenesis of PE (6). PE-related endothelial dysfunction may be associated with changes in lipid structure and oxidative stress caused by abnormal lipid metabolism (25, 26). Physiological hyperlipidemia, which occurs during normal pregnancy, increases the energy supply to both mothers and fetuses. Once the concentrations of TG and free fatty acid in pregnant women gradually increase beyond the physiological tolerance ranges of normal pregnancy, lipid molecules could accumulate and cause extensive vascular endothelial damage and systemic inflammation, eventually leading to PE (25). Consistent with previous reports, we found a significant increase of TC and TG in PE patients in early pregnancy compared with controls, which provides further evidence for abnormal lipid metabolism as a susceptibility factor for PE.

*APOBEC3A* belongs to the apolipoprotein B mRNA editing enzyme and catalytic polypeptide-like protein family, catalyzing cytidine deamination in single-stranded DNA/RNA to uridine (27). It plays an essential role in the host’s innate immune by inducing viral mutations to protect cells from viral infection (28). Apart from this, *APOBEC3A*-induced genomic mutations may drive the progression of multiple cancers (29). In this study, we identified a deletion CNV including *APOBEC3A*, *APOBEC3A_B*, and *APOBEC3B* in the genomes of controls (44%). In contrast, the incidence of this CNV is lower in PE patients (10%), which may lead to elevated levels of both *APOBEC3A* and *APOBEC3B* in PE patients. *APOBEC3A_B* has the promoter and coding region of *APOBEC3A* and the 3’UTR of *APOBEC3B*. The resulting chimeric *APOBEC3A* transcription product would lead to a further increase in intracellular *APOBEC3A* levels (30). Subsequently, we also confirmed by qPCR that the copy numbers of *APOBEC3A* and *APOBEC3A_B* genes were markedly higher in PE patients than in controls. Notably, our findings showed that the copy number of *APOBEC3A* was statistically positively correlated with serum concentrations of TC and LDL. *LPA* is known to be associated with metabolic diseases (31) and may be involved in the pathogenesis of PE (14–17). *BTNL3* has also been reported to have a possible role in intestinal metabolism and inflammation in neonates with intrauterine growth restriction (32). Our study found that copies of both *LPA* and *BTNL3* were significantly and positively associated with HDL levels. Moreover, *in vivo* and *in vitro* studies suggested that *APOBEC3A* may regulate cholesterol and fatty acid metabolisms. These findings filled the gap in the role of *APOBEC3A* in regulating lipid metabolisms. Of note, we found that *APOBEC3A* expression was also increased in patients with non-alcoholic steatohepatitis (NASH), especially in NASH in combination with fibrosis (unpublished data), suggesting that *APOBEC3A* may play a role in other metabolic diseases as well. Overall, elevated *APOBEC3A* copies may lead to increased cholesterol and fatty acid biosynthesis and decreased catabolism, which increases the risks of various metabolic diseases. These findings may guide the dietary management and clinical use of drugs in such high-risk groups and have good clinical application prospects, although the specific mechanism by which *APOBEC3A* regulates lipid metabolism needs in-depth study.

PE is a gestational disease, and the main manifestations in animal models include elevated blood pressure and urinary protein, restricted fetal growth, elevated sFlt-1, decreased PLGF, and increased sFlt-1/PLGF ratio (33). In the present study, we found that the average fetal weight per pregnant mice overexpressing *APOBEC3A* was distinctly reduced (Figure 5F), a manifestation of growth restriction. Both sFlt-1 levels and sFlt-1/PLGF ratios were distinctly increased in the placenta (Figure 5G). However, changes in plasma parameters were not evident (Supplementary Figure 4), which led us to speculate that to be due to vascular endothelial damages may be only related to local (placenta) changes in PE rather than systemic ones. On the other hand, PE is a complex disease induced by multiple factors where a single element may be difficult to interpret the pathogenesis of PE. Our molecular and clinical data suggest that aberrant *APOBEC3A* copies could at least partly explain the pathogenesis of PE.

In terms of limitations, we did not conduct a subgroup analysis of the collected cases as early-onset and late-onset PE due to limited recruitment, despite the pathogenesis of PE may differ among patients in those two subtypes. Further, our recruited participants were all from one city in China, as PE patients from other regions were not available at the time of this study. This study retrospectively collected information on the lipid profile of pregnant women in early gestation, which resulted in a certain percentage of missing data. We did not account for the effect of possible confounding factors, such as familial dyslipidemia and the use of lipid-lowering drugs, on this study. In addition, as the blood pressure of mice may be affected by various conditions such as sounds, light, and temperature, we did not measure the blood pressure of mice in this study. Finally, *in vivo* and *in vitro* knockout models need to be constructed to explore further the more detailed mechanisms that *APOBEC3A* may modulate lipid metabolism and participate in the pathogenesis of PE.

Collectively, this study established a novel link between genetics and lipid metabolism in the development of PE. That is, aberrant copies of *APOBEC3A* may be involved in the pathogenesis of PE by regulating lipid metabolism. This critical finding will likely facilitate a better understanding of the molecular mechanisms of PE. It is also our understanding that *APOBEC3A* copies and lipid profiles may potentially have clinical applications to benefit the early identification of pregnant women at high risk of PE, leading to early clinical intervention to reduce the risk of developing PE.

## Data Availability Statement

The original contributions presented in the study are included in the article/Supplementary Material, further inquiries can be directed to the corresponding author.

## Ethics Statement

The studies involving human participants were reviewed and approved by The Medical Research Ethics Committee of International Peace Maternity and Child Health Hospital. The patients/participants provided their written informed consent to participate in this study. The animal study was reviewed and approved by The Institutional Animal Care and Use Committee of Shanghai Institute of Materia Medica, Chinese Academy of Sciences.

## Author Contributions

NL, L-KG, and B-SW conceived and designed the experiments. NL, ML, and J-HS performed the experiments. Y-NG, X-JW, JM, XZ, YC, and J-HL provided clinical materials. NL, Y-TH, FZ, HH, and J-LX collected and analyzed the data. NL wrote the manuscript. L-KG and B-SW reviewed and edited the manuscript. All authors read and approved the submitted version of the manuscript.

## Conflict of Interest

The authors declare that the research was conducted in the absence of any commercial or financial relationships that could be construed as a potential conflict of interest.

## Publisher’s Note

All claims expressed in this article are solely those of the authors and do not necessarily represent those of their affiliated organizations, or those of the publisher, the editors and the reviewers. Any product that may be evaluated in this article, or claim that may be made by its manufacturer, is not guaranteed or endorsed by the publisher.

## Footnotes

1
http://dgv.tcag.ca/dgv/app/home

## Abbreviations

AAV: Adeno-associated Virus
CNVs: copy number variations
FPKM: fragment of the exon model per million mapped reads
GO: Gene Ontology
HDL: high-density lipoprotein
LDL: low-density lipoprotein
PE: preeclampsia
PLGF: placental growth factor
qPCR: quantitative polymerase chain reaction
sFlt-1: soluble fms-like tyrosine kinase-1
SNVs: single nucleotide variants
TC: total cholesterol
TG: triglyceride.
