# Supplementary material for: Copy Number Analyses Identified a Novel Gene: APOBEC3A Related to Lipid Metabolism in the Pathogenesis of Preeclampsia
Source: Front Cardiovasc Med. 2022 May 16;9:841249. doi: 10.3389/fcvm.2022.841249 (PMC9149004; doi:10.3389/fcvm.2022.841249)
Supplement: Supplementary file 1 [file Data_Sheet_1.DOCX]

Supplementary Material

# Supplementary Tables

## Table S1. The quality control analysis of the microarrays

| **Sample** | **Threshold Test** | **SexCall (Biology)** | **MAPD≤0.25** | **SNPQC≥15.00** | **Waviness SD≤0.12** |
| --- | --- | --- | --- | --- | --- |
| Con_1 | Within Bounds | female | Pass | Pass | Pass |
| Con_2 | Within Bounds | female | Pass | Pass | Pass |
| Con_3* | Within Bounds | male | Pass | Pass | Pass |
| Con_4 | Within Bounds | female | Pass | Pass | Pass |
| Con_5 | Within Bounds | female | Pass | Pass | Pass |
| Con_6 | Within Bounds | female | Pass | Pass | Pass |
| Con_7 | Within Bounds | female | Pass | Pass | Pass |
| Con_8 | Within Bounds | female | Pass | Pass | Pass |
| Con_9 | Within Bounds | female | Pass | Pass | Pass |
| Con_10 | Within Bounds | female | Pass | Pass | Pass |
| PE_1 | Within Bounds | female | Pass | Pass | Pass |
| PE_2 | Within Bounds | female | Pass | Pass | Pass |
| PE_3 | Within Bounds | female | Pass | Pass | Pass |
| PE_4 | Within Bounds | female | Pass | Pass | Pass |
| PE_5 | Within Bounds | female | Pass | Pass | Pass |
| PE_6 | Within Bounds | female | Pass | Pass | Pass |
| PE_7 | Within Bounds | female | Pass | Pass | Pass |
| PE_8 | Within Bounds | female | Pass | Pass | Pass |
| PE_9 | Within Bounds | female | Pass | Pass | Pass |
| PE_10 | Within Bounds | female | Pass | Pass | Pass |

*The sample was judged as male by the ChAS software with unclear reasons, and the sample was excluded from subsequent analyses. PE, preeclampsia.

## Table S2. Primer sequences for qPCR analysis.

| **Gene** | **Primer** | **Sequence (5'→3')** |
| --- | --- | --- |
| *GAPDH* | FW | AAGAAGGTGGTGAAGCAGG |
|  | RV | AGGTGGAGGAGTGGGTGTCG |
| *BTNL3* | FW | TTACTCTGTGGTTGCCCTGTG |
|  | RV | ACACTCCCACATACCACCCT |
| *LPA* | FW | CTTGGATTGAGGGAATGATGAGA |
|  | RV | CCTTACCCACGTTTCAGCTTCTA |
| *LMF1* | FW | ATGCTTGGAGCAGGCCTGA |
|  | RV | TTGCACTTGAACTCGTAGTCCTCC |
| *APOBEC3A* | FW | ATGGAAGCCAGCCCAGCATCC |
|  | RV | CAGGCGCTCCACTTCGTAGCA |
| *APOBEC3B* | FW | TCCAGACACATTCACTTTCAA |
|  | RV | AGAAAGCCCATGTGCTGGTCC |
| *APOBEC3A_B* | FW | TGCGCCTCAGCCTCCTCTTT |
|  | RV | GTCTCTCCTCCGATGGTACC |

## Table S3. Primer sequences for RT-PCR analysis in humans.

| **Gene** | **Primer** | **Sequence (5'→3')** |
| --- | --- | --- |
| *GAPDH* | FW | CTGGGCTACACTGAGCACC |
|  | RV | AAGTGGTCGTTGAGGGCAATG |
| *APOBEC3A* | FW | TGGCATTGGAAGGCATAAGAC |
|  | RV | TTAGCCTGGTTGTGTAGAAAGC |
| *FDPS* | FW | TGTGACCGGCAAAATTGGC |
|  | RV | GCCCGTTGCAGACACTGAA |
| *DGAT2* | FW | AGCAGGTGATCTTCGAGGAG |
|  | RV | CATGGGGCGAAACCAATGTA |
| *SCD* | FW | TCTAGCTCCTATACCACCACCA |
|  | RV | TCGTCTCCAACTTATCTCCTCC |
| *FASN* | FW | AAGGACCTGTCTAGGTTTGATGC |
|  | RV | TGGCTTCATAGGTGACTTCCA |
| *CD36* | FW | AAAATGGGCTGTGACCGGAA |
|  | RV | TCCAAACACAGCCAGGACAG |
| *GPD1* | FW | GCCATCTGAAGGCAAACGC |
|  | RV | GCCAATGGTTGTCTCACAGAAC |
| *CPT1A* | FW | TCCAGTTGGCTTATCGTGGTG |
|  | RV | TCCAGAGTCCGATTGATTTTTGC |

## Table S4. Primer sequences for RT-PCR analysis in mice.

| **Gene** | **Primer** | **Sequence (5'→3')** |
| --- | --- | --- |
| *Gapdh* | FW | TCTCCTGCGACTTCAACA |
|  | RV | TGGTCCAGGGTTTCTTACT |
| *Hmgcr* | FW | AGCTTGCCCGAATTGTATGTG |
|  | RV | TCTGTTGTGAACCATGTGACTTC |
| *Hmgcs2* | FW | GAAGAGAGCGATGCAGGAAAC |
|  | RV | GTCCACATATTGGGCTGGAAA |
| *Fdps* | FW | GGAGGTCCTAGAGTACAATGCC |
|  | RV | AAGCCTGGAGCAGTTCTACAC |
| *Srebp1* | FW | GATGTGCGAACTGGACACAG |
|  | RV | CATAGGGGGCGTCAAACAG |
| *Fasn* | FW | GGAGGTGGTGATAGCCGGTAT |
|  | RV | TGGGTAATCCATAGAGCCCAG |
| *Acc1* | FW | GATGAACCATCTCCGTTGGC |
|  | RV | GACCCAATTATGAATCGGGAGTG |
| *Adiporq* | FW | TGTTCCTCTTAATCCTGCCCA |
|  | RV | CCAACCTGCACAAGTTCCCTT |
| *Acoxl* | FW | AAGTCCCTCTGTATAGATTGGCA |
|  | RV | CCTTGGCAGACAACACACTC |

## Table S5. Clinical characteristics of the participants for CNV genotyping.

| **Variables** | **PE patients (n=10)** | **Controls (n=10)** | **P value** |
| --- | --- | --- | --- |
| **In early pregnancy (12 to 14 gestational weeks)** | | | |
| Body mass index, kg/m2 | 23.72 (3.18) | 22.46 (3.41) | 0.404 |
| SBP, mmHg | 111.00 (11.25) | 107.50 (17.00) | 0.625 |
| DBP, mmHg | 72.40 (6.17) | 71.40 (8.06) | 0.759 |
| TC, mmol/L | 4.92 (0.50) | 4.62 (0.34) | 0.133 |
| TG, mmol/L | 1.41 (0.75) | 1.35 (0.60) | 0.841 |
| HDL, mmol/L | 1.67 (0.42) | 1.64 (0.36) | 0.898 |
| LDL, mmol/L | 2.87 (0.55) | 2.40 (0.71) | 0.114 |
| **At diagnosis** | | | |
| Age, years | 30.30 (3.06) | 30.90 (3.73) | 0.698 |
| SBP, mmHg | 144.70 (9.57) | 118.30 (7.66) | <0.001 ^a^ |
| DBP, mmHg | 88.90 (9.54) | 71.80 (6.81) | <0.001 ^a^ |
| Gestational age at delivery, week | 37.67 (0.81) | 38.93 (0.91) | 0.004 ^a^ |
| Proteinuria, g/24h | 0.62 (0.45) | 0.05 (0.02) | <0.001 ^a^ |
| Fetal weight, g | 2882.00 (464.76) | 3253.00 (468.14) | 0.092 |

Abbreviations: PE, preeclampsia; SBP, systolic blood pressure; DBP, diastolic blood pressure; TC, total cholesterol; TG, triglyceride; HDL, high-density lipoprotein; LDL, low-density lipoprotein.

Variables were expressed as mean (SD). The p values were calculated by the unpaired two-tailed Student's t-test.

^a^ p≤0.05.

## Table S6. Differential genes linked to abnormal CNVs.

| **Chromosome** | **Segment** | **Gene** | **DGV** | **Style** | **Length/kb** | **Difference*/ %** |
| --- | --- | --- | --- | --- | --- | --- |
| 9 | p24.1 | *SPATA6L* | No | Loss | 10.79 | 40 |
| X | p22.33 | *PPP2R3B* | No | Loss | 151.699 | 38 |
| 8 | q13.2 | *PREX2* | No | Loss | 4.316 | 30 |
| X | q26.2 | No | No | Loss | 3.702 | 30 |
| X | p22.31 | No | Yes | Loss | 18.87 | -67 |
| X | p21.3 | *IL1RAPL1* | Yes | Loss | 2.642 | -56 |
| 7 | p22.3 | No | Yes | Gain | 38.04 | -44 |
| 11 | p15.4 | *OR52N5, OR52N1* | Yes | Loss | 25.449 | -44 |
| 15 | q26.3 | *CHSY1* | Yes | Gain | 4.011 | -44 |
| 17 | q21.31 | No | Yes | Loss | 16.019 | -44 |
| 7 | q36.2 | *DPP6* | Yes | Loss | 16.388 | -38 |
| 14 | q32.33 | No | Yes | Loss | 30.509 | -37 |
| 17 | q21.2 | *KRTAP9-7* | Yes | Gain | 9.738 | -37 |
| 1 | q23.1 | *NTRK1* | Yes | Loss | 5.739 | -36 |
| 5 | q35.3 | *BTNL3* | Yes | Loss | 52.036 | -34 |
| 6 | q26 | *LPA* | Yes | Gain | 14.633 | -34 |
| 17 | q12 | *TBC1D3, TBC1D3F, LOC440434* | Yes | Loss | 126.753 | -34 |
| 20 | q13.12 | *EYA2* | Yes | Loss | 11.981 | -34 |
| 20 | q13.33 | No | Yes | Loss | 3.664 | -34 |
| 22 | q13.1 | *APOBEC3A, APOBEC3A_B, APOBEC3B* | Yes | Loss | 39.416 | -34 |
| 3 | p14.2 | *FHIT* | Yes | Loss | 7.283 | -33 |
| 8 | q24.11 | *EXT1* | Yes | Loss | 3.497 | -33 |
| 10 | q26.11 | No | Yes | Loss | 4.095 | -33 |
| 16 | p11.2 | *PRR14, FBRS* | Yes | Loss | 16.785 | -33 |
| 16 | p13.3 | *LMF1* | Yes | Gain | 1.409 | -33 |
| 18 | q21.32 | *MALT1* | Yes | Loss | 2.3 | -33 |
| 20 | q13.2 | *BCAS1, MIR4756* | Yes | Loss | 5.135 | -33 |

*****This difference refers to the frequency of the CNV in PE patients minus the frequency in normotensive pregnant women.

# Supplementary Figures


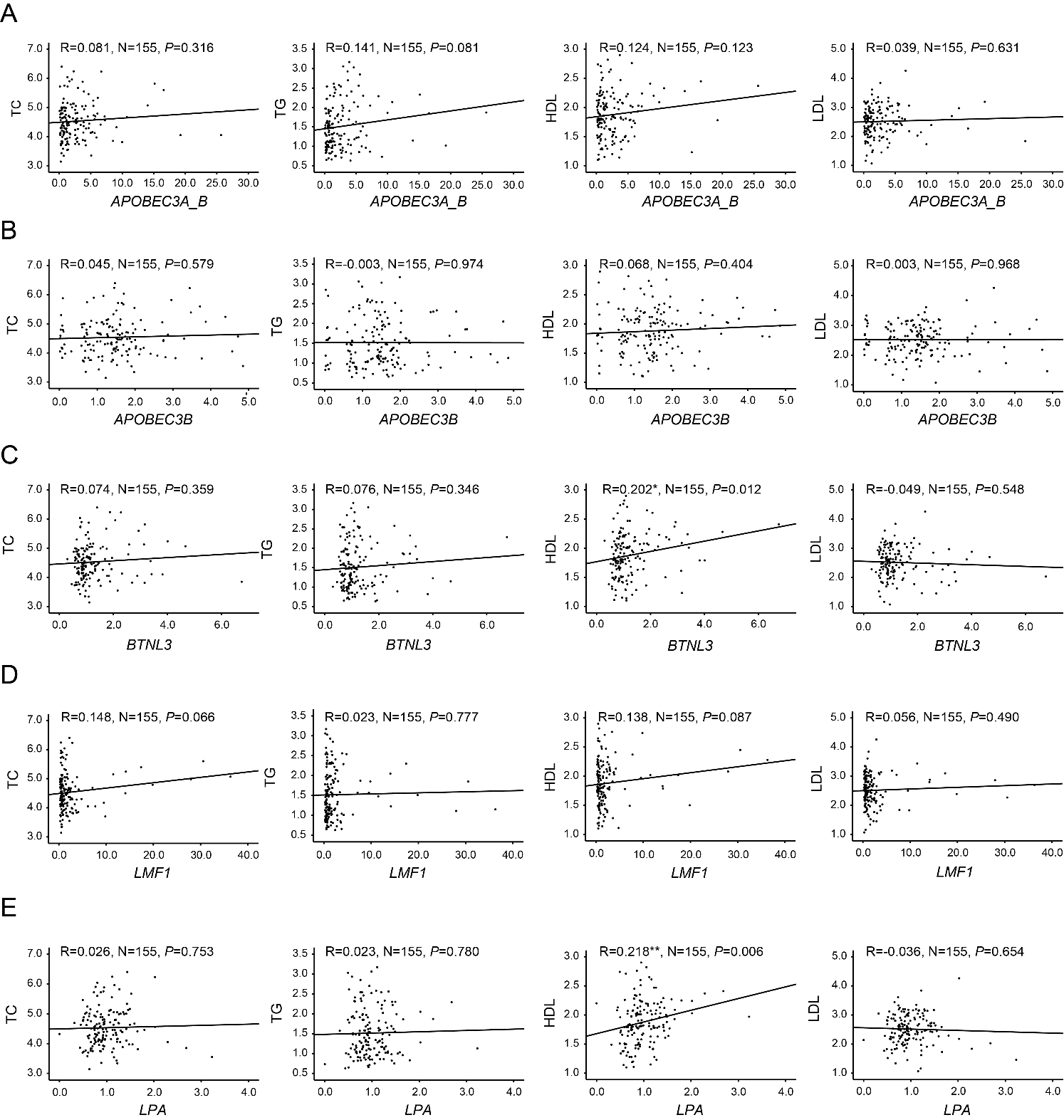


**Supplementary Figure 1.** Correlation between clinical lipid profiles and the copy numbers of candidate genes. Correlations between the copy number of (A) *APOBEC3A_B*, (B) *APOBEC3B*, (C) *BTNL3*, (D) *LMF1*, and (E) *LPA* and clinical lipid profiles (n=155). The correlations were analyzed by the Pearson correlation coefficient. ** p<0.01, * p<0.05.


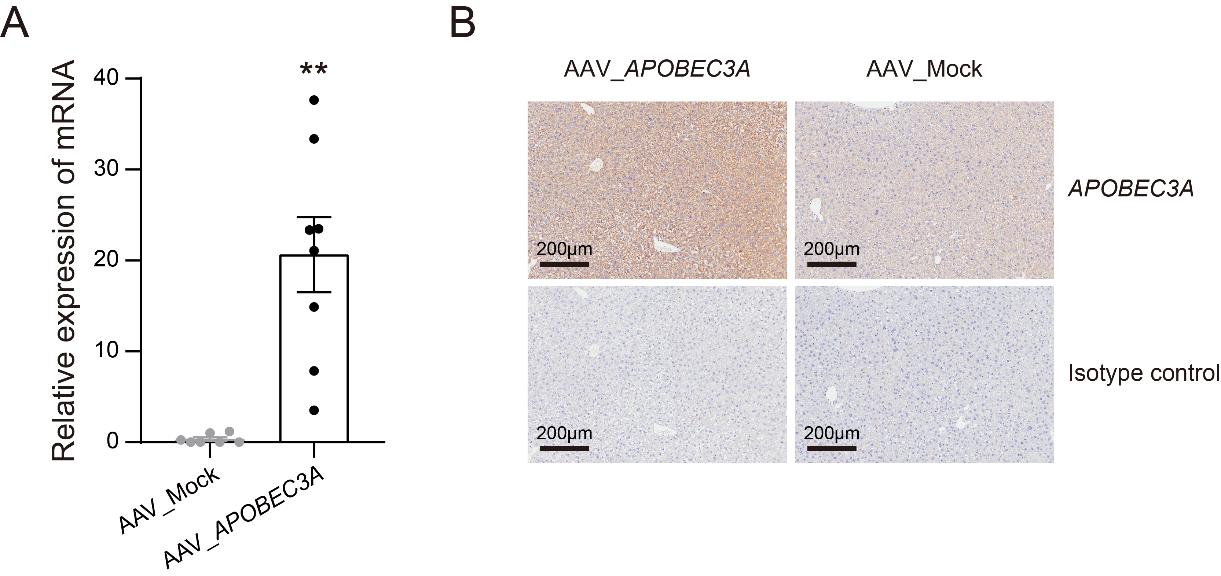


**Supplementary Figure 2.** Hepatic-specific overexpression of *APOBEC3A* in pregnant mice. The (A) mRNA and (B) protein expression of *APOBEC3A* gene in the liver of pregnant mice injected with AAV_*APOBEC3A* or AAV_Mock (n=8/group). The unpaired two-tailed Student's t-test determined statistical significances between two groups. Data were represented as the mean ± SEM. ** p<0.01.


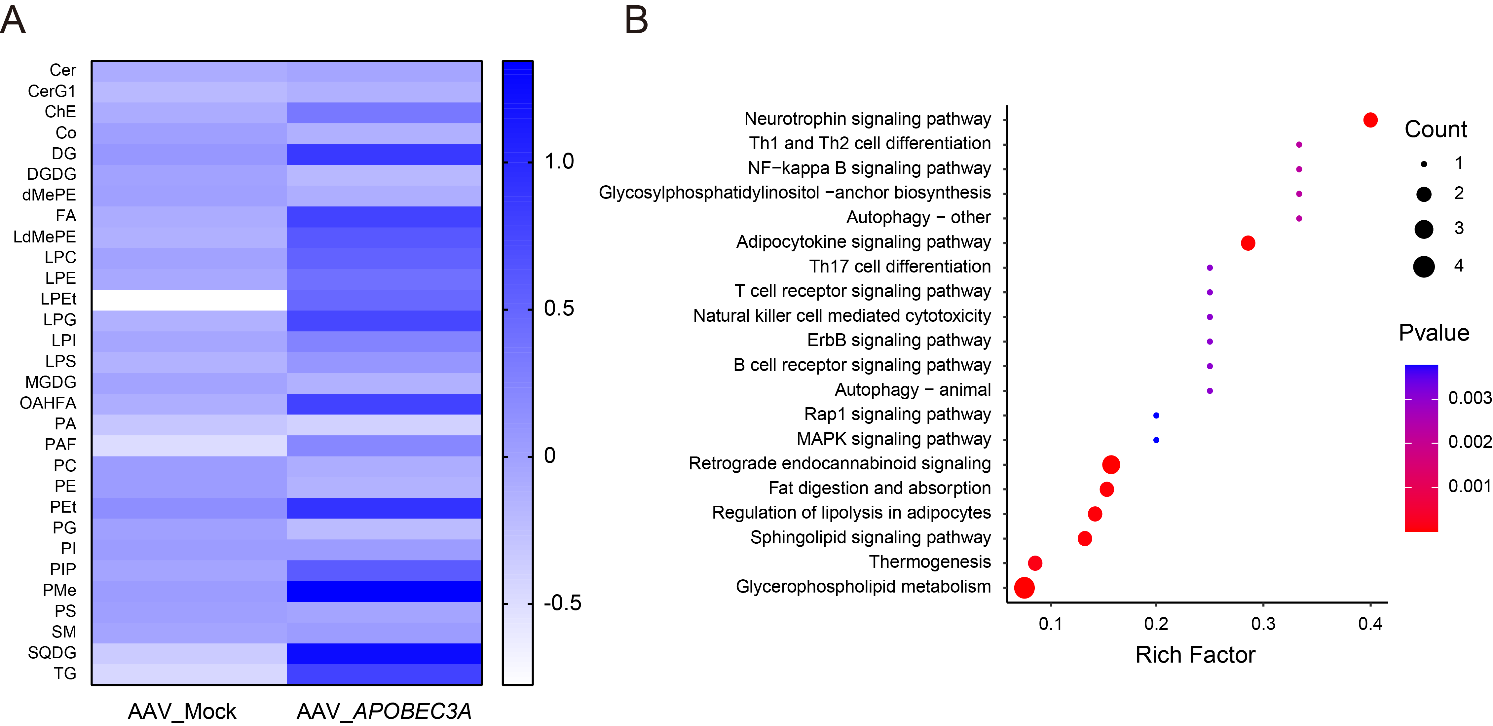


**Supplementary Figure 3.** Lipid metabolites in AAV_*APOBEC3A* and AAV_Mock. (A) Differential lipid metabolites in the AAV_*APOBEC3A* and AAV_Mock groups (n=6/group). (B) Differential lipids were enriched in glycerophospholipid metabolism, adipocytokine signaling pathway, fat digestion and absorption, regulation of lipolysis in adipocytes, and cholesterol metabolism. Cer, ceramides; CerG1, monogylcosylceramide; chE, cholesteryl ester; Co, coenzyme; DG, diacylglycerol; DGDG, digalacrosyldiacylglycerol; dMePE, dimethylphosphatidylethanolamine; FA, fatty acids; LdMePE, lyso-dimethylphosphatidylethanolamine; LPC, lyso-phosphatidylcholine; LPE, lyso-phosphatidylethanolamine; LPEt, lyso-phosphatidylethanol; LPG, lyso-phosphatidylglycerol; LPI, lyso-phosphatidylinositol; LPS, lyso-phosphatidylserine; MGDG, monogalacrosyldiacylglycerol; OAHFA, (O-acyl)-1-hydroxy fatty acids; PA, phosphatidic acid; PAF, platelet-activating factor; PC, phosphatidylcholine; PE, phosphatidylethanolamine; PEt, phosphatidylethanol; PG, phosphatidylglycerol; PI, phosphatidylinositol; PIP, phosphatidylinositol phosphate; PMe, phosphatidylmethanol; PS, phosphatidylserine; SM, sphingomyelin; SQDG, sulfoquinovosylmonoacylglycerol; TG, triglycerides.


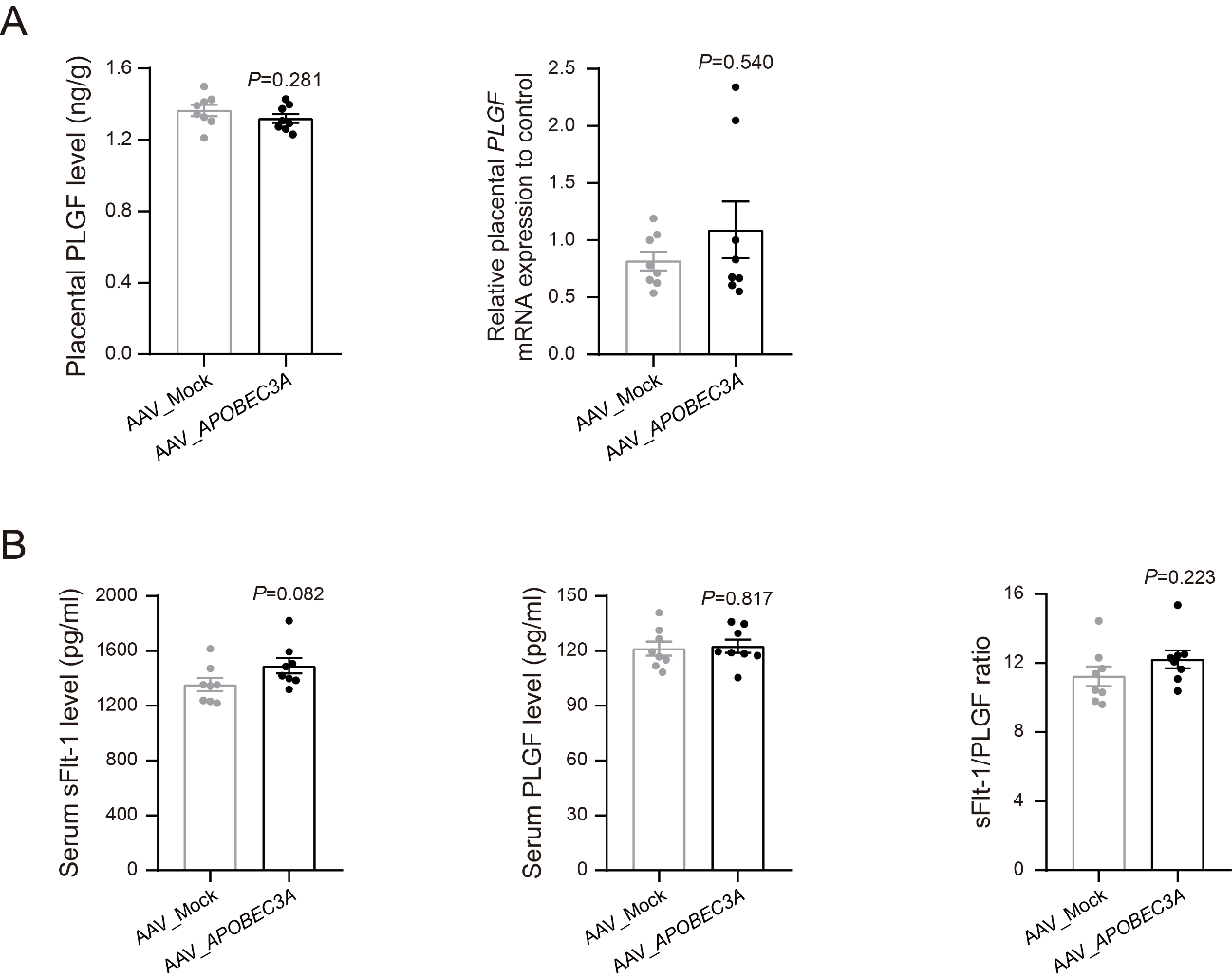


**Supplementary Figure 4.** Levels of sFlt-1 and PLGF in placenta and plasma of pregnant mice overexpressing *APOBEC3A*. (A) The protein and mRNA levels of PLGF in the placenta of pregnant mice injected with AAV_*APOBEC3A* or AAV_Mock (n=8/group). (B) The levels of sFlt-1, PLGF, and sFlt-1/PLGF ratio in the serum of pregnant mice injected with AAV_*APOBEC3A* or AAV_Mock (n=8/group). The unpaired two-tailed Student's t-test determined statistical significances between two groups. Data were represented as the mean ± SEM.
